# Prevalence and Predictors of Renal Disease in a National Representative Sample of the Romanian Adult Population: Data from the SEPHAR IV Survey

**DOI:** 10.3390/diagnostics12123199

**Published:** 2022-12-16

**Authors:** Călin Pop, Oana Florentina Gheorghe Fronea, Ioana Antonia Branea, Lucian Mihai Itu, Roxana Darabont, Irinel Parepa, Theodora Benedek, Maria Dorobantu

**Affiliations:** 1 Emergency Clinical County Hospital of Baia Mare, 430130 Baia Mare, Romania; 2 Faculty of Medicine Arad, Str. Feleacului nr. 1, 310414 Arad, Romania; 3 Faculty of Medicine, University of Medicine and Pharmacy “Carol Davila”, Bulevardul Eroii Sanitari nr. 8, Sector 5, 050474 Bucuresti, Romania; 4 Cardiology Department, Clinical Emergency Hospital Bucharest, Bulevardul Eroii Sanitari nr. 8, Sector 5, 014461 Bucuresti, Romania; 5 Department of Mathematics and Computer Science, Transilvania University of Brasov, B-dul Eroilor nr. 29, 500036 Brașov, Romania; 6 Department of Automation and Information Technology, Transilvania University of Brasov, B-dul Eroilor nr. 29, 500036 Brașov, Romania; 7 “Ovidius” State University Constanta, Faculty of Medicine, Cardiology Dept, Campus Aleea Universitatii nr. 1, 900470 Constanta, Romania; 8 Cardiology Department, County Clinical Emergency Hospital, University of Medicine and Pharmacy, Street Gh. Marinescu, 38, 540142 Targu Mures, Romania

**Keywords:** prevalence, chronic kidney disease, hypertension, uric acid levels

## Abstract

Background: The prevalence of chronic kidney disease (CKD) correlates with the prevalence of hypertension (HT). We studied the prevalence and predictors of CKD in a representative sample of the Romanian adult population. Methods: A sample of 1470 subjects were enrolled in the SEPHAR IV (Study for the Evaluation of Prevalence of Hypertension and Cardiovascular Risk) survey. All subjects were evaluated for blood pressure (BP) and extensive evaluations of target organ damage, blood, and urine samples were undertaken. Results: A total of 883 subjects were included in the statistical analysis. Those experiencing CKD with an eGFR < 60 mL/min/1.73 m^2^ were older at 71.94 ± 7.4 years (n = 19, 2.15%) compared with those without renal impairment at 50.3 ± 16.21 years (n = 864, 97.85%), *p* < 0.0001. The prevalence of CKD among hypertensives (379 from 883) was 4.49% (17/379), while 17 out of 19 subjects with CKD had HT (89.47%). After adjusting for age, sex, and diabetic status, only serum uric acid (SUR) > 6.9 mg/dL (OR: 6.61; 95% CI: 2.063, 10.83; *p* = 0.004) was an independent risk factor and a predictor of CKD. Conclusions: The prevalence of CKD in hypertensive Romanian adults was more than ten times higher than in the normotensive population. Levels of SUR > 6.9 mg/dL were predictors of CKD.

## 1. Introduction

The incidence and prevalence of CKD are increasing worldwide, along with the burdens of HT, diabetes mellitus (DM), hyperlipidemia, obesity, smoking, and aging. Patients with CKD are at a high risk of progressing toward end-stage renal disease (ESRD) and early morbidity and mortality due to premature cardiovascular disease (CVD). As a result, the increasing number of CKD patients is becoming a common and significant public health problem that leads to poor quality of life and demands expensive health resources [1,2,3,4,5]. CKD represents an important challenge in low- and middle-income countries, which are not well-equipped to deal with its consequences. The Global Burden of Chronic Kidney Disease Collaboration report showed that in 2017, the global prevalence of CKD was 9.1%, or approximately 700 million cases. The prevalence of CKD has increased by 29.3% (26.4 to 32.6, 95% CI) since 1990; however, the age-standardized prevalence remained unchanged during this period (1.2%, −1.1 to 3.5, 95% CI). The mortality from CKD increased in the same period by 41.5% (35.2 to 46.5, 95% CI), while the age-standardized mortality remained unchanged, with a 2.8% change (−1.5 to 6.3, 95% CI) from 1990 to 2017. CKD is the 12th leading cause of death globally as of 2017, with a 4.6% (4.3 to 5.0) rate of global deaths [6]. The prevalence and mortality concerning CKD present differential variations between countries, with major increases and recognitions in those where the prevalence of DM and HT are increasing. These two risk factors have had divergent trajectories: while DM prevalence has increased and global blood pressure has declined in high-income and middle-income countries, both have remained unchanged or even increased in the majority of low- and middle-income countries [7,8]. Strategies for reducing the cardiovascular risk of CKD should be adopted in each country; however, there is an unmet need for further evaluation in clinical trials, particularly in patients with advanced CKD or ESRD. The high number of cases and the significant pluriorganic impact of CKD justify enhanced efforts for better prevention and treatment. A low serum creatinine-based estimated glomerular filtration rate (eGFR) and raised urinary albumin measured using the urinary albumin-to-creatinine ratio (ACR) are markers of the severity of CKD [9]. Therefore, the classification of CKD is based on eGFR and ACR, and the most commonly used system is the Kidney Disease Improving Global Outcomes classification [10]. Romania, as previously shown in the three-national representative surveys (SEPHAR I (2005), SEPHAR II (2012), and SEPHAR III (2016)—Study for the Evaluation of Prevalence of Hypertension and Cardiovascular Risk in Romania), is a high-cardiovascular-risk Eastern European country with a high prevalence of HT at approximately 45.1%, which, together with its complications, leads to 62% of total deaths [11,12,13,14]. The SEPHAR III survey was conducted in 2016 and revealed an increasing trend in the prevalence of DM (17.8%), obesity (47%), and dyslipidemia (83.2%) with subclinically and clinically overt target organ damage (TOD) as follows: albuminuria 30–300 mg/g in 4.9%, albuminuria > 300 mg/g in 2.2%, eGFR: 30–60 mL/min/1.73 m^2^ in 4.6%, eGFR < 30 mL/min/1.73 m^2^ in 0.7%, heart failure (HF) in 15.3%, coronary artery disease (CAD) in 32.1%, atrial fibrillation (AF) in 8.1%, stroke in 5.1%, peripheric arterial diseases (PAD) in 3.7%, and carotid plaques in 22.5%. Therefore, 82% of hypertensive patients in the SEPHAR III survey were at high or very high risk. Most (72.2%) of the hypertensive subjects were treated with two or more drugs (51.9%), but only 30.8% of them had controlled BP values [14,15,16]. In this context, a new survey, SEPHAR IV (Study for the Evaluation of Prevalence of Hypertension and Cardiovascular Risk in Romania, 4th Edition), was conducted between May and July 2020 to confirm the estimated trend of the prevalence of HT, TOD, and significant cardiovascular risk factors. Therefore, this study aimed to assess the prevalence and predictors of CKD among adult subjects that attended the SEPHAR IV survey.

## 2. Materials and Methods

A detailed description of the SEPHAR IV methodology was previously published; therefore, we present below only those aspects regarding the collection of SEPHAR IV data that were the object of this study [17].

### 2.1. SEPHAR IV: Sample Selection and Data Collection

The sample population of SEPHAR IV consisted of 1470 adult subjects (18–80 years) who provided informed consent for participation in this new survey and were enrolled from the eight territorial regions of Romania according to the National Statistics Institute, resulting in a nationally representative sample for the Romanian adult population (the minimum number of individuals for representativity was 1379 adult subjects). In each of the eight regions, the fieldwork was carried out by a study team of four members: one senior cardiologist, two young cardiologist specialists, and one study nurse. They received specific training concerning the completion of the questionnaire and the correct anthropometric, BP, and arterial rigidity measurements, including proper echocardiographic image acquisition. All the nurses received specific training regarding the collection and transportation of biological samples. The fieldwork was performed in a particular medical caravan, namely, the SEPHAR BUS, which was fully equipped with the medical and laboratory equipment required for the clinical, laboratory, and echocardiographic assessments and traveled to each of the study sites according to the regional schedule of the survey. During the two study visits, in the morning during a four-day interval (like previous SEPHAR surveys), all enrolled individuals were evaluated using the following: 51-item survey questionnaire (11 items regarding socio-demographic data; 10 items regarding medical history and cardiovascular risk factors; 15 items regarding awareness of cardiovascular prevention and complications due to poor control of risk factors, as well as usage of preventive methods; 1 item regarding medication; 6 items regarding the Morisky Medication Adherence Scale; 8 items regarding the Epworth Sleepiness Scale) and anthropometric and BP measurements, together with evaluation for target organ damage, blood, and urine sample collection after a proper fasting time. The study protocol and its implementation procedures were supervised by the project reviewers and approved by the National and Local Ethics Committee (approval response nr 5S/4 from 24 September 2019).

### 2.2. Blood Pressure Measurement and Definition

The BP measurement technique and definitions of HT were in accordance with the current European Society of Hypertension (ESH) guidelines [18]. During each study visit, three BP measurements were performed at one-minute intervals using an automatic, certified BP measuring device with an adjustable cuff for arm circumferences from 24 to 42 cm. The same devices were used throughout the survey and in all the geographic regions. BP values were calculated using the arithmetic mean of the second and third BP measurements of each study visit. BP was measured in orthostatic positions at one minute and three minutes to check for orthostatic hypotension. 

The classification of optimal, normal blood pressure (NBP) and high blood pressure (HBP) was performed in accordance with the current ESH guidelines [18]. HBP was defined as systolic blood pressure (SBP) of at least 140 mmHg and/or diastolic blood pressure (DBP) of at least 90 mmHg at both visits and previously diagnosed HT under antihypertensive treatment during the previous two weeks, regardless of BP values. Controlled SBP values were defined as less than 140 mmHg and controlled DBP values were defined as less than 90 mmHg at both study visits in hypertensive patients who were receiving antihypertensive treatment.

### 2.3. Risk Factors and Diagnostic Categories

The SEPHAR IV questionnaire included items dedicated to HT-related comorbidities: coronary heart disease (CHD); heart failure (HF); atrial fibrillation (AF); transient ischemic attack (TIA); stroke; peripheral artery disease (PAD); and renal failure (RF), including the need for dialysis. The 12-lead ECG recording was performed with standard electrode placement with the patient in a supine position, standing still, and breathing normally. Standard transthoracic echocardiography two-dimensional, color and spectral Doppler was performed with the patients in the left lateral decubitus position, ECG-gated, with image acquisitions over three cardiac cycles to measure the following parameters: the thickness of the interventricular septum, the thickness of the left ventricular (LV) posterior wall, the LV mass, the LV ejection fraction, the transmitral E/A ratio, the left atrial area, and the left atrial volume. Echocardiographic measurements were performed at a workstation by two independent operators. Arterial stiffness was assessed using an oscillometer (Arteriograph TensioMed) with the subject at rest in a supine position on the dominant arm. The following parameters were measured: aortic pulse wave velocity, aortic augmentation index, aortic systolic BP, aortic pulse pressure, reverse time, systolic area index, diastolic area index, ejection duration, and diastolic reflection area. The standard ankle–brachial index measurements (ABI) were assessed using a Doppler pencil and an adequately sized BP cuff. The ABI for each subject was the smallest between the right ABI and the left ABI. Blood samples were drawn by the nurse after checking the proper fasting time before the test (between 8 and 14 h). The samples were collected while the subject was sitting. The following laboratory tests were performed: total cholesterol (TC), HDL-cholesterol (HDL-C), LDL-cholesterol (LDL-C), triglycerides (TG), apolipoprotein B (ApoB), blood glucose, glycated hemoglobin (HbA1c), serum creatinine, serum uric acid (SUR), albuminuria, urinary sodium, urinary creatinine, and urine albumin-to-creatinine ratio. 

We considered a patient as having CKD if they were diagnosed and documented in their medical history as having CKD or if they fulfilled the definition criteria at the time of the survey. CKD is defined as an eGFR < 60 mL/min/1.73 m^2^ (GFR categories G3a–G5) and one or more markers of kidney damage: albuminuria (albumin excretion rate (AER) > 30 mg/24 h, ACR > 30 mg/g (>3 mg/mmol)), urine sediment abnormalities, electrolytes, other abnormalities due to tubular disorders, abnormalities detected using histology, structural abnormalities detected using imaging, and a history of kidney transplantation [10]. The 2021 CKD-EPI equation is now the recommended standard, which is expressed as a single equation with serum creatinine as the substrate: eGFR_cr_ = 142 × min(S_cr_/κ, 1)^α^ × max(S_cr_/κ, 1)^−1.200^ × 0.9938^Age^ × 1.012 (if female) where: S_cr_ is the standardized serum creatinine in mg/dL, κ = 0.7 (female) or 0.9 (male), α = −0.241 (female) or −0.302 (male), min(S_cr_/κ, 1) is the minimum of S_cr_/κ or 1.0, max(S_cr_/κ, 1) is the maximum of S_cr_/κ or 1.0, and age is given in years [19,20]. Proteinuria was classified according to the laboratory result if a patient had an AER > 30 mg/24 h or ACR > 30 mg/g (>3 mg/mmol) [20]. DM was defined as self-reported diabetes or the use of hypoglycemic agents or both. The BMI (body mass index) is calculated by dividing a person’s weight in kilograms by their height in meters squared: normal weight is considered a BMI of 18.5–24.9 kg/m^2^, overweight is considered a BMI of 25–29.9 kg/m^2^, and obese is considered a BMI of ≥30 kg/m^2^ [21].

### 2.4. Statistical Analyses

Statistical analysis was performed using the SciPy.Stats and Statsmodel Python packages. A significant level of *p* ≤ 0.05 was considered. Descriptive analyses (means, standard deviation, range for continuous data, and proportions analysis for categorical data (95% confidence interval)) were performed for all the target variables. Student’s *t*-test was used to compare the means, and the chi-squared test or Fisher’s exact test was used to compare proportions where appropriate. We conducted a logistic regression analysis to study the predictors of CKD after adjusting for age, sex, and DM for statistically significant variables after a univariate analysis. We used a *k* × *k* confusion matrix to identify and describe the performance of the regression algorithm.

## 3. Results

A total of 1470 adults were enrolled in the study with their signed, written, informed consent. Among the total participants, 883 had completed renal laboratory tests and were included in the statistical analysis for the CKD study. 

### 3.1. Socio-Demographic Characteristics

Out of the total subjects included in the analysis (883), more than half (523, 59.23%) were female. The mean age was 60.9 ± 10.12 years, with a significant difference between those with CKD with an estimated GFR < 60 mL/min/1.73 m^2^ (71.94 ± 7.4 years) and those with an estimated GFR ≥ 60 mL/min/1.73 m^2^ (50.3 ± 16.21 years) (*p* < 0.0001). Of the participants, 341 (38.5%) were rural residents, and more than half (503, 56.96%) had low or medium levels of education. Those with high levels of education were predominantly present in the group with an estimated GFR ≥ 60 mL/min/1.73 m^2^ (43.52%, *p* = 0.08), while those with low or medium levels of education were more frequent in the group with CKD (78.95%, *p* = 0.08). All the results are shown in Table 1.

### 3.2. Prevalence of CKD

Using the CKD-EPI equation, an estimated GFR ≥ 60 mL/min/1.73 m^2^ was reported among 864 (97.85%) subjects, while an estimated GFR < 60 mL/min/1.73 m^2^, which characterized the CKD pattern, was reported in 19 (2.15%) (refer to Table 1). Stages 3a, 3b, 4, and 5 were present in 15 (1.7%), 3 (0.34%), 1 (0.11%), and 0 subjects, respectively (refer to Table 2). The prevalence of CKD among hypertensive subjects (379 from 883) was 4.49% (17/379), while 17 out of 19 subjects with CKD had HT (89.47%). Of those hypertensives with CKD, 13 out of 17 were present in stage 3a (76.47%), 3 in stage 3b (17.64%), and 1 in stage 4 (5.88%). The prevalence of CKD in the normotensive population was 0.39% (2/504 subjects). The values for creatinine, albuminuria, ACR, and SUR were significantly higher in those with CKD, while no difference was found for urinary sodium (refer to Table 1). 

### 3.3. Study Groups’ Clinical Characteristics and Related Comorbidities

Out of 883 subjects, 379 (42.93%) were hypertensives, with a greater HT prevalence among subjects with CKD (17, 89.47% compared with those with an estimated GFR ≥ 60 mL/min/1.73 m^2^ (362, 41.9%) (*p* < 0.0001). The new hypertensives (68, 7.87%) were found exclusively in the group of subjects with an estimated GFR ≥ 60 mL/min/1.73 m^2^ (refer to Table 1). Controlled hypertensives (172, 47.51%) were more frequent in the group with an estimated GFR ≥ 60 mL/min/1.73 m^2^ compared with the 5 (29.41%) in the CKD group (*p* = 0.04) (refer Table 3). Those experiencing DM, obesity, AF, HF, diastolic dysfunction, increased LV mass, and carotid plaques were significantly more frequent among subjects with CKD. Based on clinical and laboratory data, the prevalence of metabolic syndrome (MS) was statistically the highest among those with CKD: 62.8% vs. 31.5%. Furthermore, a TG/HDL-C ratio > 3.0 was significantly more prevalent in CKD patients: 68.8% vs. 3.25%. Additionally, higher levels of SUR (7.44 ± 1.51 mg/dL) were present in those with CKD compared with 5.26 ± 1.57 mg/dL in subjects without renal impairment (refer to Table 1).

### 3.4. Treatment and Control of Hypertension

The treatment and control of hypertension are exhibited in Table 3. The mean SBP of 127.46 ± 17.44 mmHg was significantly lower among subjects with an estimated GFR ≥ 60 mL/min/1.73 m^2^ compared with 139.32 ± 19.45 mmHg in the subjects with CKD (*p* = 0.003). The inhibitors of the renin–angiotensin–aldosterone system (RASi), diuretics, calcium channel blockers (CCB), beta blockers (βB), aldosterone antagonists (AA), tritherapy, and more than three antihypertensive drugs were significantly more commonly used among subjects with CKD. The lipid-lowering drugs and antiplatelets were also significantly more commonly used among these subjects. 

### 3.5. Determinants of CKD

**Figure 1:**
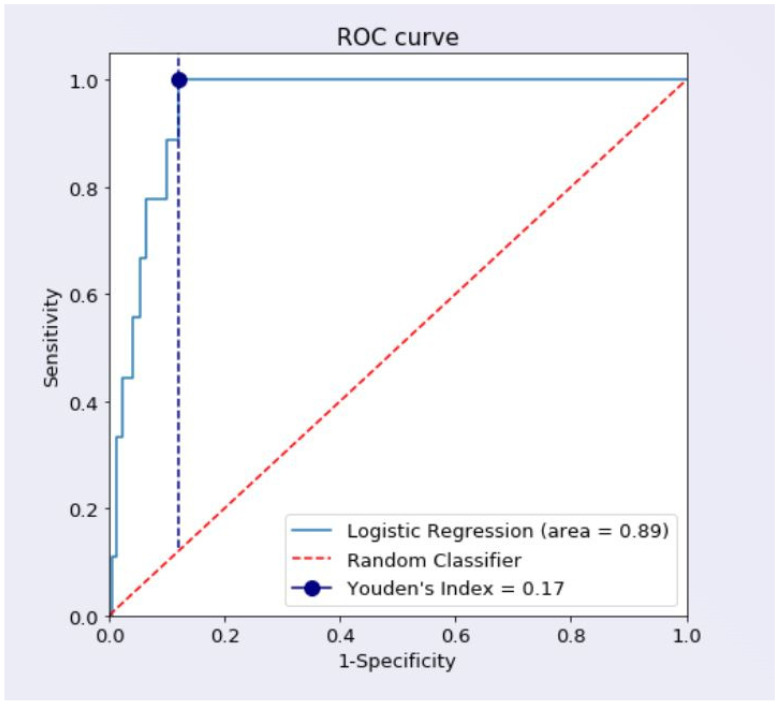
ROC curve that plots the trade-off between the sensitivity and specificity of the logistic model.

The determinants of CKD are shown in Table 4. In the univariate analysis, age > 50 years (OR: 1.12; 95% CI: 0.57, 3.84; *p* = 0.07), female sex (OR: 1.16; 95% CI: 0.4, 1.42; *p* = 0.03), SUR > 6.9 mg/dL (OR: 6.71; 95% CI: 2.72, 9.52; *p* = 0.0004), ACR > 30 mg/g (OR: 1.43; 95% CI: 1.02, 3.34; *p* = 0.05), DM (OR: 2.8; 95% CI: 2.08, 7.08; *p* = 0.03), HbA1c % > 7.5 (OR: 2.1; 95% CI: 1.93, 4.87; *p* = 0.04), LV mass > 140 g (OR: 1.46; 95% CI: 0.39, 3.63; *p* = 0.02), presence of carotid plaques (OR: 2.2; 95% CI: 0.84, 4.1; *p* = 0.001), obesity (OR: 1.23; 95% CI: 1.40–1.98; *p* = 0.04), AF (OR: 1.42; 95% CI: 0.34, 2.63; *p* = 0.01), and HF (OR: 1.4; 95% CI: 0.54, 2.8; *p* = 0.04) were associated with the risk of developing CKD. After the multivariable analysis (adjusting for age, sex, and DM status), only SUR > 6.9 mg/dL (OR: 6.61; 95% CI: 2.063, 10.83; *p* = 0.004) and the presence of carotid plaques as a marker surrogate of atherosclerosis (OR: 2.09; 95% CI: 1.1, 2.31; *p* = 0.07) were found to be independent risk factors of CKD. Hyperuricemia patients with SUR > 6.9 mg/dL had a significantly lower eGFR by an average of 12.18 mL/min/m^2^ compared with normal uricemia patients (59.15 versus 71.33 mL/min/m^2^, *p* < 0.001). One could observe that the regression and confusion matrix algorithm using SUR > 6.9 mg/dL as an independent variable correctly predicted 88.72% of patients with eGFR ≥ 60 and 88.89% of those with eGFR < 60 mL/min/1.73 m^2^. In the ROC curve that plots the trade-off between the sensitivity (=0.99) and specificity (=0.17) of the model (refer to Figure 1), one can notice the proximity between the perfect model and our logistic regression. The predicted accuracy of the regression was 89% overall, representing the area under the ROC curve. Moreover, Youden’s Index (=0.17) was used in finding the cutoff point where the vertical distance between the diagonal line (random classifier) and the ROC curve (logistic regression) was the maximum.

## 4. Discussion

To the best of our knowledge, our research is among the few epidemiological studies on CKD prevalence in the general Romanian population [22,23]. It showed that 2.15% of hypertensive and normotensive adults experienced CKD, while stages 3a, 3b, 4, and 5 were present in 1.7%, 0.34%, 0.11%, and 0% of subjects, respectively. There was a decline in the prevalence of CKD compared with the SEPHAR III survey in the overall population, which was mostly attributable to the decline in the prevalence of late CKD stages. Specifically, the overall prevalence of CKD stages 3 to 5 was 4.6% in the SEPHAR III survey (2015–2016) and 1.7% in SEPHAR IV. Similarly, the prevalences of eGFR < 30 mL/min/1.73 m^2^ were 0.7% and 0.11%, respectively; refer to Table 2 [15]. These levels were significantly lower than those previously reported in Romania and compared with other countries in 2012 and 2015: 7.32% in a study of 60,969 Romanian adults (CKD stages 3–5 prevalences ranged from 5.96% to 1.35%); 6.74% in another study with a sample of 2717 adults; and 3.31% to 17.3%, respectively, (CKD stages 3–5 prevalence ranged from 1.0% to 5.9%) in a survey of the general European population [22,23,24,25]. However, our research was the first Romanian study that aimed to describe CKD prevalence among hypertensive subjects. The prevalence of CKD was much higher among hypertensive subjects at 4.49% (17/379), while stages 3a, 3b, 4, and 5 were present in 76.47% (15/19), 17.64% (3/19), 5.88% (1/19), and 0% of subjects (refer to Table 2). Therefore, the prevalence among Romanian hypertensives was lower than those generally reported worldwide: the pooled prevalence of CKD was 17.8% in a meta-analysis from Africa (2021), the prevalence of CKD among hypertensive US adults was 35.8% in a survey from 2011 to 2014, 7.6% to 61.2% in Asian populations (2021), 4.2% to 14.3% across European countries (2016), 18.8% in Spain among hypertensives older than 60 years, and 6.7% in hypertensives taking antihypertensive medication in the Czech Republic [25,26,27,28,29,30]. There were no new patients with CKD among the new hypertensives discovered in our study, and those with CKD were known to be hypertensive and already treated, while 17 out of 19 subjects with CKD had HT (89.47%). Like the Czech results, the low prevalence of CKD in hypertensive individuals could be explained by better BP control among those using medication. These changes were accompanied by the increased use of RASi and CCBs in hypertensives, along with the increased use of RAS inhibitors in those with CKD [30]. Of note, approximately 62.7% of CKD hypertensive individuals were using either RAS inhibitors, CCBs, or both, whereas only 51.09% of hypertensive individuals without renal damage were using these drugs in our study. As shown in Table 3, RASi, diuretics, CCB, βB, AA, tritherapy, and more than three antihypertensive drugs were significantly used among subjects with CKD. However, the proportion of controlled hypertensives in the CKD group (5, 29.41%) was not satisfactory, and the mean SBP of 139.32 ± 19.45 mmHg was significantly higher compared with 127.46 ± 17.44 mmHg observed in subjects with an estimated GFR ≥ 60 mL/min/1.73 m^2^ (*p* = 0.003). It should be noted that the prevalence of HT was lower compared with the SEPHAR III survey (42.93% prevalence and 7.87% newly diagnosed HT versus 45.1% prevalence and 19.1% newly diagnosed HT), and the proportion of hypertensive individuals achieving BP control was higher: 46.7% versus 30.8% [14]. We found a higher prevalence of CKD among males and populations with low-level education (refer to Table 1). This was consistent with other observations regarding the higher prevalence and renal impact of HT in these subjects [31,32,33]. Finally, our research recruited around 40% of targeted hypertensive and normotensive subjects who participated four years earlier in a previous survey (SEPHAR III), possibly explaining the difference in CKD prevalence with the positive effect of self or institutionally implemented preventive strategies after the SEPHAR III survey [14,15,16]. The values of creatinine, albuminuria, ACR, and SUR as an expression of affected renal hemodynamics were significantly higher in those with CKD, while no difference was found in urinary sodium (refer to Table 3). Comorbidities such as DM, obesity, AF, HF, diastolic dysfunction, increased LV mass, and carotid plaques were significantly more frequent among subjects with CKD (refer to Table 1). This was in accordance with the pathophysiological implications for renal damage, especially in hypertensive patients, and with the very high cardiovascular risk observed in CKD patients [1,2,34,35]. In our study, more patients with CKD had dyslipidemia and used lipid-lowering drugs (42.11% vs. 14.93%), largely due to increased triglyceride levels, decreased HDL-C, and varying levels of LDL-C (refer to Table 1 and Table 3). CKD likely contributed to dyslipidemia, and this association was multifactorial and mediated by inflammation and oxidative stress, leading together to atherosclerosis and a higher risk of death from cardiovascular disease [36]. In a recent study, the monocyte-count-to-HDL-cholesterol ratio (MHR) emerged as a reliable biomarker of inflammation and oxidative stress in CKD. The MHR seems to be linked with multiple antihypertensive drugs, which characterize resistant hypertension and are inversely related to eGFR. Unfortunately, the study design did not permit us to calculate the MHR for our CKD patients [37]. MS (we use the definition of the International Diabetes Federation consensus) is a clinical and metabolic condition that includes many risk factors for CKD. [38]. As in our study, many reports showed an association between the increasing number of MS components and the risk and prevalence of CKD [39,40]. As a result, when managing risk factors in CKD patients, all components of metabolic syndrome should be considered. The TG/HDL-C ratio can be used to predict insulin resistance (IR), as well as metabolic and cardiovascular disease risk [41,42]. A value > 3 is associated with IR and increased cardiometabolic risk and, like MS, it was more frequently seen among CKD patients in our study (refer to Table 1). Therefore, these findings are a reconfirmation of the high cardiovascular risk of CKD patients and the need for preventive strategies to reduce cardiovascular morbidity and mortality. In the univariate analysis, age > 50 years, female sex, SUR > 6.9 mg/dL, ACR > 30 mg/g, DM, HbA1c % > 7.5, LV mass > 140 g, presence of carotid plaques, obesity, AF, and HF were associated with the risk of developing CKD. There are contradictory details on these relationships, but growing evidence suggests that there are correlations that could be bidirectional between these biomarkers and the cardiovascular clinical and subclinical manifestations and renal damage [43,44,45,46,47,48,49,50,51,52,53,54]. From the multivariate analysis, after adjusting for age, sex, and DM status, only SUR > 6.9 mg/dL (OR: 6.61; 95% CI: 2.063, 10.83; *p* = 0.004) and the presence of carotid plaques as a marker surrogate of atherosclerosis (OR: 2.09; 95% CI: 1.1, 2.31; *p* = 0.07) were independent risk factors of CKD. In our study, those with CKD had higher levels of SUR (7.44 ± 1.51 mg/dL) compared with those without renal damage (5.26 ± 1.57 mg/dL; 95% CI 5.15, 5.49; *p* = 0.0001), and a significantly lower eGFR by an average of 12.18 mL/min/m^2^ compared with normal uricemia patients, that is, 59.15 versus 71.33 mL/min/m^2^, *p* < 0.001. This observation concorded with the already published data from the SEPHAR III survey and other studies, wherein the results revealed the association between hyperuricemia and incident chronic kidney disease [55,56]. The mechanism of hyperuricemia and kidney impairment is a matter of debate despite the strong correlation between SUR and the renal resistive index [48]. Some experimental data suggested that uric-acid-induced kidney damage is due to the enhanced activation of the RAS and the reduced endothelial nitric oxide availability [57]. Additionally, hyperuricemia is frequently encountered in patients with HT and metabolic syndrome, both of which are classic risk factors for CKD [49]. Other studies demonstrated that higher levels of SUR (the normal upper limit is 6.8 mg/dL, and any symptoms can occur over 7 mg/dL) may be a major contributor to the development or progression of CKD. Although there is no clear cutoff for the SUR value associated with the risk of kidney damage, there appears to be an increased risk as uric acid levels rise [55,56,57,58,59,60,61,62]. While our logistic regression logistic model used SUR > 6.9 mg/dL as an independent variable, it correctly predicted 88.72% of patients with eGFR ≥ 60 and 88.89% of those with eGFR < 60 mL/min/1.73 m^2^; however, the validity precludes due to the limited number of sample size and absence of follow-up. An important clinical and prognostic question in this context is whether there is a recommendation for prescribing a urate-lowering drug (ULD) for CKD individuals. None of the subjects had a ULD, such as the xanthine oxidase inhibitor allopurinol, prescribed for them during the study. Our subjects were asymptomatic, while gout is present in 25% and hyperuricemia in 60% of patients with CKD. Controversy persists over whether prescribing a ULD is warranted in CKD patients, especially in those with asymptomatic hyperuricemia. In 2020, two large randomized clinical trials (RCTs) found no benefit of allopurinol in slowing renal progression [63,64]. The ALLHEART study included older patients (>age 60) with a history of ischemic heart disease and no gout who were randomized into allopurinol or placebo groups and followed for five years without any cardioprotective effect [65]. In a more recent meta-analysis of 16 RCTs involving 1943 patients, a prescribed ULD (allopurinol, febuxostat, and benzbromarone) was not found to have a renoprotective effect compared with a placebo. Febuxostat lowered urate better than allopurinol and was superior to a placebo at controlling BP, while no differences were observed with allopurinol and benzbromarone. Moreover, febuxostat is slightly superior to allopurinol in lowering the decline in eGFR and the increase in proteinuria, but the difference did not reach statistical significance [66]. If the clinical arguments are strong enough to recommend a ULD in symptomatic subjects (the goal would be to lower SUR < 6 mg/dL), for those who are asymptomatic, there is an unmet need to develop a carefully designed RCT to determine the role of uric acid in the progression of CKD [67]. The presence of carotid atherosclerotic plaques is well recognized as a marker of atherosclerosis, which in patients with CKD, plays a role in the development of CV disease and could enhance the progression of renal disease [51]. Even if the results in our study have only borderline statistical significance due to the limited sample size, we found more carotid plaques among subjects with CKD (refer to Table 1). Different studies showed that the prevalence of CKD is significantly associated with the presence of carotid plaques after adjustment for confounding risk factors, especially in those with calcified, echolucent, or unstable characteristics [52,68,69,70,71].

### Study Strengths and Limitations

The strengths of this study included the nationally representative population-based samples that covered both urban and rural areas of Romania; the wide range of demographic, clinical, and laboratory data; and the rigorous and standardized methods used for the screenings and data collection. Among the total participants (1470), 883 had completed renal laboratory tests and were included in the statistical analysis. However, the population studied was sufficiently represented and the statistical analysis was adequate. The major limitations of the study were the single measurement of corresponding variables (which could impact the SUR and CKD definitions), the limited sample of those with complete renal data, and the cross-sectional design that limited the interpretation of determinants and causal relationships.

## 5. Conclusions

In Romanian adults, there was a decline in the prevalence of CKD that was attributable to the decline in the prevalence of HT, which was related to better control of known hypertensives. The prevalence of CKD in Romanian adults with hypertension was more than ten times higher than in the normotensive population, while 89.47% of subjects with CKD experienced HT. Levels of SUR > 6.9 mg/dL were predictors of CKD. Screening for hyperuricemia in hypertensives may play a role in identifying patients at risk of developing CKD. Our data provide important information regarding the trend in the prevalence of hypertension-related CKD and may guide the further implementation of specific preventive strategies in a country with a high cardiovascular risk. 

## Figures and Tables

**Table 1:** Characteristics of the hypertensive and non hypertensive individuals according to eGFR CKD-EPI ≥ 60, or <60 mL/min/1.73 m^2^. Legend: eGFR—estimated glomerular filtration rate, BMI—body mass index, MS—metabolic syndrome, ACR—urinary albumin-to-creatinine ratio, TC—total cholesterol, LDL—low density lipoprotein cholesterol, HDL—high density low cholesterol, TG—trylicerides, Apo B—apolipoprotein B, EFLV—ejection fraction of left ventricle, LV—left ventricle, IV—intraventricular, DM—diabetes mellitus, AF—atrial fibrillation, MI—myocardial infarction, HF—heart failure, N—numbers, %—percentage, *p* ≤ 0.05, CI—confidence interval (95% confidence level).

Variables	Overall	eGFR ≥ 60 mL/min/1.73m^2^	eGFR < 60 mL/min/1.73 m^2^	*p*	CI (95% Confidence Level)
Total subjects (N, %)	883 (100%)	864 (100%)	19 (100%)	<0.0001	(0.96, 0.98)
Known Hypertensives (N, %)	311 (35.22%)	294 (34.03%)	17 (89.47%)	<0.0001	(0.75, 0.84)
New Hypertensives (N, %)	68 (7.7%)	68 (7.87%)	0 (0%)	0.402	(1, 1)
Total hypertensives	379 (42.93%)	362 (41.9%)	17 (89.47%)	<0.0001	(0.93, 0.97)
Controlled hypertensives	87 (9.85%)	82 (9.49%)	5 (26.32%)	0.041	(0.89, 0.99)
Age (years)	60.9 ± 10.12	50.3 ± 16.21	71.94 ± 7.4	<0.0001	(50.39, 52.09)
Male (N, %)	360 (40.7%)	350 (40.51%)	10 (52.63)	0.1611	(0.95, 0.98)
Female (N, %)	523 (59.23%)	514 (59.49%)	9 (47.37%)	0.053	(0.97, 0.99)
High level education (N, %)	380 (43.03%)	376 (43.52%)	4 (21.05%)	0.0850	(0.97, 0.99)
Low/medium level education (N, %)	503 (56.96%)	488 (56.48%)	15 (78.95%)	0.08520	(0.95, 0.98)
Rural residence (N, %)	341 (38.5%)	330 (38.19%)	11 (57.89%)	0.1319	(0.94, 0.98)
Smoker (N, %)	232 (26.27%)	230 (26.62%)	2 (10.53%)	0.1891	(0.97, 1)
Non or past smoker (N, %)	650 (73.61%)	633 (73.26%)	17 (89.47%)	0.1859	(0.96, 0.98)
Waist (cm)	96.35 ± 16.51	96.08 ± 16.54	108.37 ± 9.01	0.0013	(95.25, 97.44)
BMI (kg/m^2^)	29.038 ± 3.24	28.22 ± 5.61	30.76 ± 4.6	0.0507	(28.54, 29.16)
MS	284 (32.16%)	272 (31.5%)	12 (62.8%)	0.001	(0.91, 0.98)
Creatinine (mg/dL)	0.91 ± 0.12	0.79 ± 0.16	1.407 ± 0.38	<0.0001	(0.79, 0.81)
Acid uric (mg/dL)	5.76 ± 1.61	5.26 ± 1.57	7.44 ± 1.51	0.0001	(5.15, 5.49)
Albuminuria (mg/dL)	12.01 ± 50.12	11.86 ± 54.24	16.63 ± 6.93	0.0474	(8.18, 15.34)
ACR (mg/g)	14.25 ± 186.24	9.19 ± 19.07	58.1 ± 218.2	<0.0001	(7.81, 13.04)
Urinary sodium (mmol/L)	120.81 ± 53.72	120.4 ± 53.6	122.7 ± 53.3	0.8952	(115.89, 124.46)
Blood glucose (mg/dL)	102.09 ± 20.12	99.89 ± 22.94	124.52 ± 42.9	<0.0001	(98.56, 101.69)
Glycated hemoglobin %	5.72 ± 0.96	5.62 ± 0.78	6.36 ± 1.49	<0.0001	(5.58, 5.68)
TC (mg/dL)	201.5 ± 52.1	202.1 ± 46.7	192.7 ± 62.3	0.38928	(198.42, 204.42)
LDL (mg/dL)	129.02 ± 51.2	132.6 ± 43.4	117.5 ± 57.7	0.15309	(128.88, 134.47)
HDL (mg/dL)	53.01 ± 14.1	53.87 ± 13.6	50.42 ± 16.77	0.2783	(52.73, 54.49)
TG (mg/dL)	122.24 ± 80.1	119.77 ± 79.8	152.35 ± 78.5	0.0965	(115.30, 126.02)
TG/HDL ratio > 3.0	283 (32.04)	270 (31.25%)	13 (68.8%)	0.001	(0.92, 0.97)
Apo B (g/L)	0.95 ± 0.32	0.95 ± 0.28	0.96 ± 0.368	0.8776	(0.93, 0.97)
EFLV ≤ 50 % (N, %)	145 (16.42%)	140 (16.2%)	5 (26.32%)	0.3876	(0.93, 0.99)
Diastolic dysfunction (N, %)	244 (27.63%)	232 (26.85%)	12 (63.16%)	0.0011	(0.92, 0.97)
Heart rate (b/min)	71.3 ± 12	72.21 ± 11.3	70.87 ± 8.6	0.6382	(71.16, 72.48)
Modified Cornell criteria > 12 mm (N, %)	685 (77.58%)	670 (77.55%)	15 (78.95%)	1.0	(0.96, 0.98)
IV troubles of conduction (N, %)	609 (68.9%)	599 (69.33 %)	10 (53.63 %)	0.1917	(0.97, 0.99)
LV mass (g)	122.6 ± 62	114.4 ± 71.7	154.67 ± 89.6	0.0238	(100.7, 110.19)
Thickness of the LV posterior wall (mm)	7.21 ± 2.2	7.41 ± 3.9	8.47 ± 4.54	0.2710	(6.57, 7.11)
Carotid plaques (N, %)	198 (22.42%)	185 (21.41%)	13 (68.42%)	<0.0001	(0.89, 0.96)
DM (N, %)	121 (13.7%)	110 (12.73%)	11 (57.89%)	<0.0001	(0.85, 0.96)
Stroke (N, %)	33 (3.74%)	31 (3.59 %)	2 (10.53%)	0.3341	(0.85, 1)
AF (N, %)	55 (6.23%)	51 (5.9%)	4 (21.05%)	0.0262	(0.85, 0.99)
MI (N, %)	21 (2.38%)	20 (2.31%)	1 (5.26%)	0.941	(0.86, 1)
HF (N, %)	44 (4.98%)	40 (4.63 %)	4 (21.05%)	0.00650	(0.82, 0.99)

**Table 2:** Stages of CKD according to the eGFR in the sample of subjects from the SEPHAR IV survey. Legend: eGFR—estimated glomerular filtration rate, G—grade, N—number, %—percentage.

Grade/Stages eGFR mL/min/1.73 m^2^	Total (N, %) 883, 100%	Urine Albumin-to-Creatinine Ratio (mg/g)	Urinary Sodium (mmol/L)
G1 ≥ 90	636 (72.03%)	7.88 ± 13.17	120.58 ± 54.59
G2 60–89	228 (25.82%)	12.98 ± 29.85	119.92 ± 50.58
G3a 45–59	15 (1.7%)	6.6 ± 5.87	124.43 ± 56.79
G3b 30–44	3 (0.34%)	324.93 ± 550.43	117.0 ± 7.0
G4 15–29	1 (0.11%)	30 ± 0.9	-
G5 < 15	0	-	-

**Table 3:** Blood pressure and exposure to drugs in the hypertensive subjects according to eGFR CKD-EPI ≥ 60 or <60 mL/min/1.73 m^2^. LEGEND: eGFR—estimated glomerular filtration rate, SBP—systolic blood pressure, DBP—diastolic blood pressure, RASi—inhibitors of the renin–angiotensin–aldosterone system, CCB—calcium channel blockers, AA—aldosterone antagonists N—number, %—percentage, *p* ≤ 0.05, CI—confidence interval (95% confidence level).

Variables	Overall (N, %)	eGFR ≥ 60 mL/min/1.73 m^2^ (N, %)	eGFR < 60 mL/min/1.73 m^2^ (N, %)	*p*	CI
Total hypertensives	379	362	17	0.009	(0.93, 0.97)
Controlled hypertensives	177 (46.7%)	172 (47.51%)	5 (29.41%)	0.04	(0.89, 0.99)
Mean SBP (mmHg)	127.7 ± 17.5	127.46 ± 17.44	139.32 ± 19.45	0.003	(126.55, 128.8)
Mean DBP (mmHg)	80.6 ± 10.53	80.65 ± 10.53	82.21 ± 10.65	0.52	(79.9, 81.37)
RASi	126 (33.24%)	120 (33.14%)	6 (35.29%)	0.06	(0.91, 0.98)
Diuretics	136 (35.88%)	126 (34.8%)	10 (58.82%)	0.002	(0.88, 0.97)
CCB	70 (18.46%)	65 (17.95%)	5 (29.41%)	0.01	(0.75, 0.91)
Beta blockers	62 (16.35%)	57 (15.74%)	5 (29.41%)	0.03	(0.9, 0.97)
AA	5 (1.31%)	4 (1.1%)	1 (5.88%)	0.02	(0.44, 1.0)
Central antihypertensive	17 (4.48%)	16 (4.41%)	1 (5.88%)	0.1	-
Monotherapy	116 (30.6%)	114 (31.49%)	2 (11.76%)	0.05	(0.93, 0.99)
Bitherapy	85 (22.42%)	81 (22.37%)	4 (23.52%)	1.0	(0.94, 1.0)
Tritherapy	54 (14.2%)	47 (12.98%)	7 (41.17%)	0.0004	(0.81, 0.96)
More than 3 antihypertensive drugs	13 (3.43%)	12 (3.31%)	1 (5.8%)	0.01	(0.94, 1.0)
Lipid-lowering drugs	137 (15.5%)	129 (14.93%)	8 (42.11%)	0.003	(0.9, 0.98)
Antiplatelets	101 (11.44%)	96 (11.11%)	5 (26.32%)	0.08	(0.9, 0.99)

**Table 4:** Predictors of CKD in patients with hypertension adjusted for sex, age, and DM. Legend: IACR—urinary albumin-to-creatinine ratio, DM—diabetes mellitus, HbA1c—hemoglobin glycated A1c, LV—left ventricle, AF—atrial fibrillation, MI—myocardial infarction, HF—heart failure, *p* ≤ 0.05, CI—confidence interval (95% confidence level), OR—odds ratio.

Variables	Univariate (Unadjusted)		Multivariate (Adjusted for Age, Sex, and Diabetes)	
	*p*-Value (CI)	OR	*p*-Value (CI)	OR
Age > 50 years	0.07 (0.57, 3.84)	1.12	0.854 (−1.48, 6.07)	0.66
Female sex	0.03 (0.4, 1.42)	1.16	0.32 (−1.46, 0.47)	0.611
Controlled hypertensives	0.22 (0.17, 2.27)	0.21	0.799 (−1.3, 1.007)	0.26
Hypertension awareness	0.31 (1.33, 4.27)	0.17	0.165 (−0.45, 2.670)	0.25
Smoker	0.666 (−0.71, 1.11)	0.82	0.395 (−0.65, 1.64)	0.64
Uric acid > 6.9 (mg/dL)	0.0004 (2.72, 9.52)	6.71	0.004 (2.063, 10.83)	6.61
ACR (mg/g)	0.05 (1.02, 3.34)	1.43	0.490 (−1.2, 6.1)	0.7
DM	0.03 (2.08, 7.08)	2.8	0.299 (−1.8, 5.8)	0.66
HbA1c % > 7.5	0.04 (1.93, 4.87)	2.1	0.40 (1.25, 5.5)	0.78
Diastolic dysfunction	0.14 (−0.45, 3.17)	0.49	0.62 (−1.5, 2.28)	0.69
LV mass > 140 g	0.02 (0.39, 3.63)	1.46	0.24 (−1.05, 2.1)	0.45
Carotid plaques	0.001 (0.84, 4.1)	2.2	0.07 (1.1, 2.31)	2.09
Stroke	0.135 (−0.35, 2.65)	0.51	0.49 (−2.2, 1.06)	0.56
Obesity	0.04 (1.40, 1.98)	1.23	0.38 (0.26, 0.47)	0.85
AF	0.01 (0.34, 2.63)	1.42	0.46 (−0.79, 1.72)	0.59
MI	0.421 (−1.21, 2.9)	0.33	0.376 (−1.17, 1.2)	0.37
HF	0.04 (0.54, 2.8)	1.4	0.465 (−0.76, 1.68)	0.57

## Data Availability

Data can be requested from the corresponding author.
